# Static and fatigue tensile properties of carbon/glass hybrid fiber-reinforced epoxy composites

**DOI:** 10.1038/s41598-022-10245-5

**Published:** 2022-04-15

**Authors:** Kimiyoshi Naito

**Affiliations:** 1 Polymer Matrix Hybrid Composite Materials Group, National Institute for Materials Science, Tsukuba, 305-0047 Japan; 2 Department of Aerospace Engineering, Tohoku University, Sendai, 980-8579 Japan

**Keywords:** Engineering, Materials science

## Abstract

The static and fatigue tensile properties of high-strength polyacrylonitrile (PAN)-based carbon (IMS60) and electronic (E)-class glass (E-glass) hybrid fiber-reinforced epoxy matrix composites (HFRPs) were investigated. The fiber orientations of the HFRP specimens were set to unidirectional with [(0_(IMS60)_)/(0_(E-glass)_)]_S_ (subscript S means symmetry and [(0_(IMS60)_)/(0_(E-glass)_)/(0_(E-glass)_)/(0_(IMS60)_)]), [(0_(E-glass)_)/(0_(IMS60)_)]_S_, [(0_(E-glass)_)/(0_(IMS60)_)_2_]_S_, [(0_(E-glass)_)/(0_(IMS60)_)_3_]_S_, [(0_(E-glass)_)/(0_(IMS60)_)_5_]_S_, [(0_(E-glass)_)_2_/(0_(IMS60)_)]_S_, [(0_(E-glass)_)_3_/(0_(IMS60)_)]_S_, and [(0_(E-glass)_)_5_/(0_(IMS60)_)]_S_. Under static loading for the [(0_(IMS60)_)/(0_(E-glass)_)]_S_, [(0_(E-glass)_)/(0_(IMS60)_)]_S_, [(0_(E-glass)_)/(0_(IMS60)_)_2_]_S_, [(0_(E-glass)_)/(0_(IMS60)_)_3_]_S_, and [(0_(E-glass)_)/(0_(IMS60)_)_5_]_S_ HFRP specimens, the stress applied to the specimen was almost linearly proportional to the strain until failure. However, the tensile stress–strain curves of the [(0_(E-glass)_)_2_/(0_(IMS60)_)]_S_, [(0_(E-glass)_)_3_/(0_(IMS60)_)]_S_, and [(0_(E-glass)_)_5_/(0_(IMS60)_)]_S_ HFRP specimens had a complicated shape (jagged trace). The Weibull statistical distributions of the tensile strength values were also examined. The Weibull moduli for the [(0_(E-glass)_)/(0_(IMS60)_)]_S_, [(0_(E-glass)_)/(0_(IMS60)_)_2_]_S_, [(0_(E-glass)_)/(0_(IMS60)_)_3_]_S_, [(0_(E-glass)_)/(0_(IMS60)_)_5_]_S_, [(0_(E-glass)_)_2_/(0_(IMS60)_)]_S_, [(0_(E-glass)_)_3_/(0_(IMS60)_)]_S_, and [(0_(E-glass)_)_5_/(0_(IMS60)_)]_S_ HFRP specimens were higher than those for the mono carbon fiber-reinforced epoxy (CFRP) and glass fiber-reinforced epoxy (GFRP) specimens. Under fatigue loading, the fatigue properties of the HFRP specimens showed CFRP-dominant behaviour at high stress levels and GFRP-dominant behaviour at low stress levels. The fatigue properties of the HFRP specimens increased with increasing volume fraction of CFRP in the following order: ([(0_(E-glass)_)/(0_(IMS60)_)_5_]_S_ > [(0_(E-glass)_)/(0_(IMS60)_)_3_]_S_ > [(0_(E-glass)_)/(0_(IMS60)_)_2_]_S_ > [(0_(IMS60)_)/(0_(E-glass)_)]_S_ > [(0_(E-glass)_)/(0_(IMS60)_)]_S_ > [(0_(E-glass)_)_2_/(0_(IMS60)_)]_S_ > [(0_(E-glass)_)_3_/(0_(IMS60)_)]_S_ > [(0_(E-glass)_)_5_/(0_(IMS60)_)]_S_).

## Introduction

Fiber-reinforced polymer matrix composites (FRPs) have become a dominant material in the aerospace, high-performance automotive, and sporting goods industries^1,2^. By mixing two or more types of fibers in a common matrix to form a hybrid composite, it may be possible to create a material possessing the combined advantages of the individual composite.

Naito et al. characterized the tensile properties and fracture behavior of high-strength polyacrylonitrile (PAN)-based and high-modulus pitch-based hybrid carbon fiber-reinforced epoxy and polyimide matrix composites (CFRPs)^3–5^. The tensile stress–strain curves of the hybrid CFRP specimens showed a complicated shape (jagged trace). The hybrid composite can be considered one example of a material that prevents instantaneous failure.

A number of papers were written approximately 1970–1980 on the advantages and applications of hybrid composites, such as carbon/glass hybrid composites, under static loading^6–10^. This interest stems from a more cost-effective utilization of expensive fiber if it is used in hybrid form^11^. The development of fiber-hybrid composites is a logical evolution toward even more design freedom and hence more possibility for optimization and cost reduction^12^. Although fatigue behavior is an important property for many applications, the effects of hybridization on this property have not been extensively studied^13^. Wu et al.^14^ reported the fatigue properties of hybrid composites. The addition of CFRP to a basalt fiber-reinforced polymer matrix composite increased the number of cycles to rupture of the hybrid composites. On the other hand, the addition of CFRP to a glass fiber-reinforced polymer matrix composite (GFRP) did not have the same effect. However, measuring the static and fatigue tensile failure of the same hybrid composites remains a challenging issue. Demonstrating the static and fatigue tensile failure of the same hybrid composites are a major original contribution of this work.

In the present work, static and fatigue tensile tests of high-strength PAN-based carbon (IMS60) and electronic (E)-class glass (E-glass) hybrid fiber-reinforced epoxy matrix composite (HFRP) specimens were performed to evaluate their potential. The Weibull statistical distributions of the static tensile strength and stiffness reduction during fatigue loading of the HFRP specimens were also evaluated.

## Experimental procedure

### Materials

HFRP laminates were produced using an epoxy matrix-based unidirectional (UD) FRP prepreg material QC133-149A (fiber: IMS60, matrix: 133) and E-glass-UD/epoxy (fiber: E-glass, matrix: 180 °C-cured-type epoxy). The IMS60 carbon fiber was a high-strength PAN-based carbon fiber, and the E-class glass fiber was an alumino-borosilicate glass fiber with less than 1% w/w alkali oxides. IMS60 (QC133-149A) prepreg was supplied by Toho Tenax Co., Ltd., and E-glass (E-glass-UD/epoxy) prepreg was supplied by Arisawa Mfg. Co., Ltd. All sheets were manufactured using conventional prepreg technology. FRP prepregs with nominal thicknesses of 0.142 mm (QC133-149A, fiber area weight (FAW): 145 g/m^2^, resin content (RC): 35%) and 0.137 mm (E-glass-UD/epoxy, FAW: 170 g/m^2^, RC: 35%) were used.

### Specimen preparation

The prepreg sheets were cut into the appropriate size and fiber orientation. The sheets were placed on a vacuum molding board. HFRP laminates were made using a hand lay-up and vacuum bagging technique (no bleeder). The fibre orientations of the mono CFRP and GFRP specimens and the HFRP specimens were set to unidirectional with (0_(IMS60)_)_4_ (subscript 4 means four layers and (0_(IMS60)_/0_(IMS60)_/0_(IMS60)_/0_(IMS60)_)), (0_(E-glass)_)_4_, [(0_(IMS60)_)/(0_(E-glass)_)]_S_ (subscript S means symmetry and [(0_(IMS60)_)/(0_(E-glass)_)/(0_(E-glass)_)/(0_(IMS60)_)]), [(0_(E-glass)_)/(0_(IMS60)_)]_S_, [(0_(E-glass)_)/(0_(IMS60)_)_2_]_S_, [(0_(E-glass)_)/(0_(IMS60)_)_3_]_S_, [(0_(E-glass)_)/(0_(IMS60)_)_5_]_S_, [(0_(E-glass)_)_2_/(0_(IMS60)_)]_S_, [(0_(E-glass)_)_3_/(0_(IMS60)_)]_S_, and [(0_(E-glass)_)_5_/(0_(IMS60)_)]_S_, respectively. The mono CFRP ((0_(IMS60)_)_4_), GFRP ((0_(E-glass)_)_4_), and the HFRP specimens ([(0_(IMS60)_)/(0_(E-glass)_)]_S_, [(0_(E-glass)_)/(0_(IMS60)_)]_S_, [(0_(E-glass)_)/(0_(IMS60)_)_2_]_S_, [(0_(E-glass)_)/(0_(IMS60)_)_3_]_S_, [(0_(E-glass)_)/(0_(IMS60)_)_5_]_S_, [(0_(E-glass)_)_2_/(0_(IMS60)_)]_S_, [(0_(E-glass)_)_3_/(0_(IMS60)_)]_S_, and [(0_(E-glass)_)_5_/(0_(IMS60)_)]_S_) were described as MC, MG, HA, HB, HC, HD, HE, HF, HG, and HH, respectively.

The fiber volume fractions of the mono CFRP and GFRP specimens and the HFRP specimens are listed in Table 1. The prepreg sheets were pressed at 490 kPa and cured at 180 °C for 4 h (the heating rate was 1 °C/min) using an autoclave (Ashida Mfg. Co., Ltd., ACA Series) in the laboratory.

**Table 1:** Volume fraction of elements for the mono CFRP and GFRP, and HFRP specimens.

	Described as	Volume fraction of IMS60 fiber *V*_*F*(*IMS60*)_ (%)	Volume fraction of E-glass fiber *V*_*F*(*E-glass*)_ (%)	Volume fraction of fiber *V*_*F*_ (%)	Volume fraction of IMS60 matrix *V*_*M*(*IMS60*)_ (%)	Volume fraction of E-glass matrix *V*_*M*(*E-glass*)_ (%)	Volume fraction of matrix *V*_M_ (%)
IMS60 CFRP (QC133-149A) (0_(IMS60)_)_4_	MC	56.7	0.0	56.7	43.3	0.0	43.3
E-glass GFRP (E-glass-UD/Epoxy) (0_(E-glass)_)_4_	MG	0.0	48.7	48.7	0.0	51.3	51.3
[(0_(IMS60)_)/(0_(E-glass)_)]_S_	HA	28.8	24.0	52.8	22.0	25.2	47.2
[(0_(E-glass)_)/(0_(IMS60)_)]_S_	HB	28.8	24.0	52.8	22.0	25.2	47.2
[(0_(E-glass)_)/(0_(IMS60)_)_2_]_S_	HC	38.2	15.9	54.1	29.2	16.7	45.9
[(0_(E-glass)_)/(0_(IMS60)_)_3_]_S_	HD	42.9	11.9	54.8	32.7	12.5	45.2
[(0_(E-glass)_)/(0_(IMS60)_)_5_]_S_	HE	47.5	7.9	55.4	36.3	8.3	44.6
[(0_(E-glass)_)_2_/(0_(IMS60)_)]_S_	HF	19.3	32.1	51.5	14.8	33.8	48.5
[(0_(E-glass)_)_3_/(0_(IMS60)_)]_S_	HG	14.5	36.2	50.8	11.1	38.1	49.2
[(0_(E-glass)_)_5_/(0_(IMS60)_)]_S_	HH	9.7	40.4	50.1	7.4	42.5	49.9

The laminates were cut into rectangular straight-side tensile test specimens with dimensions of 200 mm in length (gage length, *L*, of 100 mm) and 10 mm in width. The fiber axis in the specimen was oriented in line with the length of the tensile test specimen (0° direction specimen). To remove the effect of stress concentrations caused by surface roughness from the edges, the edges of the tensile test specimens were polished to remove scratches. Thinner plain-woven fabric glass fiber-reinforced plastic (50 mm in length, 10 mm in width, and 1 mm in thickness) tapered tabs were affixed to the tensile test specimen to minimize damage from the grips on the tensile testing machine. Similar specimen preparation procedures of other hybrid composites have been observed in the reported literature^3–5^.

### Static test

Static tests of HFRP specimens were performed using a universal testing machine (Shimadzu, Autograph AG-series) with a load cell of 50 kN. The specimen was set up in the testing machine. A crosshead speed of 5.0 mm/min was applied, and all tests were conducted under the laboratory environment at room temperature (at 23 °C ± 3 °C and 50% ± 5% relative humidity). Strain gauges were used to measure longitudinal strains. Similar static test procedures of other hybrid composites have been observed in the reported literature^3–5^. Ten specimens were tested for each individual type of specimen.

### Fatigue test

Fatigue tests of HFRP specimens were conducted using a servo-hydraulic testing machine (Servopulser EHF-E05-20L, Shimadzu) with a 50-kN load cell at a frequency of 10 Hz under cyclic loading with a constant amplitude. The waveform of the cyclic loads was sinusoidal. The stress ratio, *R*, of the minimum stress to the maximum stress was 0.1. The fatigue tests were terminated after 1 × 10^7^ cycles. All tests were conducted in the laboratory environment at room temperature (at 23 °C ± 3 °C and 50% ± 5% relative humidity). Strain gauges were used to measure longitudinal strains. Similar fatigue test procedures of other composites have been observed in the reported literature^15,16^.

## Results

### Static tensile properties

**Figure 1:**
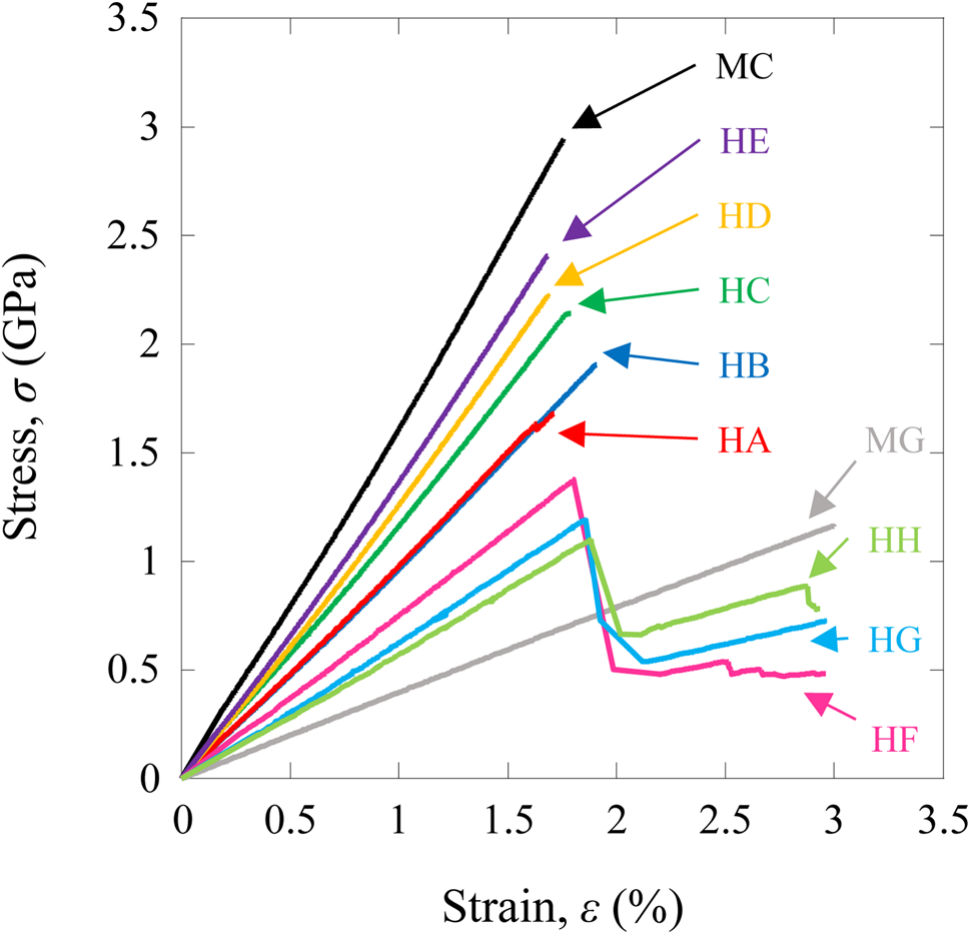
Typical tensile stress–strain curves for the CFRP, GFRP, and HFRP specimens.

Figure 1 shows typical tensile stress–strain (*σ*–*ε*) curves for the HFRP specimens, as well as for the MC and MG specimens. For the MC and MG specimens, and HA, HB, HC, HD, and HE specimens, the stress–strain response was linearly proportional until failure. However, the HF, HG, and HH specimens showed a complicated shape (jagged trace)^3–5^. For the IMS60 layers, the HFRP specimens showed an intermediate modulus in the initial stage of loading, which was taken as the tensile modulus, *E*, after which the load reached a maximum point taken as the tensile strength, *σ*_*f*_, and corresponding initial failure strain, *ε*_*f*_. Subsequently, when the IMS60 layers began to fail, the high-ductility E-glass layers held the load without instantaneous failure, exhibiting a so-called secondary tensile modulus, E* which was calculated for a constant strain range using a least square method. Finally, the load reached its secondary maximum, and fracture of the HFRP specimen occurred at the secondary fracture strength, σ*_f_, and corresponding secondary failure strain, ε*_f_. Because higher ductility E-glass fibers bear the load for a certain time after initial failure occurred, HFRP specimens with jagged traces could be considered one example of a material able to prevent instantaneous failure^3–5^. The average tensile modulus (*E*), tensile strength (*σ*_*f*_), failure strain (*ε*_*f*_), secondary tensile modulus (E*), strength (σ*_f_), and failure strain (ε*_f_) are shown in Table 2. Similar results of other hybrid composites have been observed in the reported literature^3–5^.

**Table 2:** Tensile properties of the mono CFRP and GFRP, and HFRP specimens.

	Described as	Tensile modulus *E* (GPa)	Tensile strength *σ*_*f*_ (GPa)	Failure strain *ε*_*f*_ (%)	Secondary tensile modulus E* (GPa)	Secondary fracture strength σ*_f_ (GPa)	Secondary failure strain ε*_f_ (%)	Weibull modulus *m*
IMS60 CFRP (QC133-149A) (0_(IMS60)_)_4_	MC	159 (2)	3.023 (0.168)	1.769 (0.077)	–	–	–	17.60
E-glass GFRP (E-glass-UD/Epoxy) (0_(E-glass)_)_4_	MG	38 (1)	1.109 (0.089)	2.949 (0.301)	–	–	–	12.15
[(0_(IMS60)_)/(0_(E-glass)_)]_S_	HA	96 (1)	1.747 (0.097)	1.727 (0.100)	–	–	–	17.24
[(0_(E-glass)_)/(0_(IMS60)_)]_S_	HB	96 (2)	1.996 (0.063)	1.888 (0.041)	–	–	–	29.52
[(0_(E-glass)_)/(0_(IMS60)_)_2_]_S_	HC	118 (1)	2.315 (0.077)	1.794 (0.052)	–	–	–	28.43
[(0_(E-glass)_)/(0_(IMS60)_)_3_]_S_	HD	126 (2)	2.350 (0.080)	1.713 (0.063)	–	–	–	25.65
[(0_(E-glass)_)/(0_(IMS60)_)_5_]_S_	HE	136 (3)	2.539 (0.088)	1.711 (0.056)	–	–	–	27.58
[(0_(E-glass)_)_2_/(0_(IMS60)_)]_S_	HF	76 (2)	1.559 (0.035)	1.850 (0.044)	19 (2)	0.595 (0.049)	2.969 (0.188)	42.08
[(0_(E-glass)_)_3_/(0_(IMS60)_)]_S_	HG	63 (1)	1.355 (0.032)	1.872 (0.031)	24 (1)	0.851 (0.066)	2.969 (0.135)	41.76
[(0_(E-glass)_)_5_/(0_(IMS60)_)]_S_	HH	55 (1)	1.202 (0.027)	1.933 (0.043)	28 (1)	0.992 (0.072)	2.968 (0.106)	42.35

(–) indicate standard deviation.

### Fatigue tensile properties

**Figure 2:**
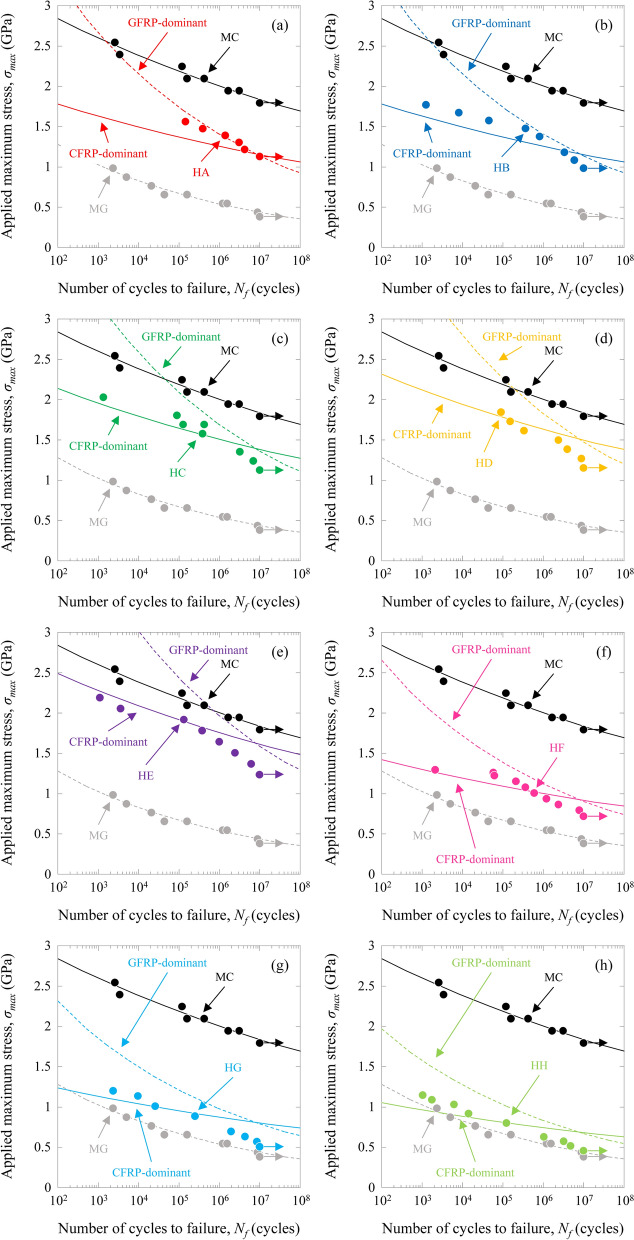
Relation between the applied maximum stress and the number of cycles to failure, *S*–*N* curves, for the HFRP specimens. (**a**) HA ([(0_(IMS60)_)/(0_(E-glass)_)]_S_), (**b**) HB ([(0_(E-glass)_)/(0_(IMS60)_)]_S_), (**c**) HC ([(0_(E-glass)_)/(0_(IMS60)_)_2_]_S_), (**d**) HD ([(0_(E-glass)_)/(0_(IMS60)_)_3_]_S_), (**e**) HE ([(0_(E-glass)_)/(0_(IMS60)_)_5_]_S_), (**f**) HF ([(0_(E-glass)_)_2_/(0_(IMS60)_)]_S_), (**g**) HG ([(0_(E-glass)_)_3_/(0_(IMS60)_)]_S_), and (**h**) HH ([(0_(E-glass)_)_5_/(0_(IMS60)_)]_S_).

Figure 2 shows the relation between the applied maximum stress, *σ*_*max*_, and the number of cycles to failure, *N*_*f*_, also defined as the *S*–*N* curves for the HFRP specimens. The *S*–*N* curves for the MC and MG specimens are also shown in this figure. For the MC and MG specimens, the fatigue properties of the MC were ~ 2–4 times higher than those of the MG. The fatigue properties of the HFRP specimens showed CFRP-dominant behaviour at high stress levels and GFRP-dominant behaviour at low stress levels. The fatigue properties of the HFRP specimens increased with increasing volume fraction of CFRP (HE > HD > HC > HA > HB > HF > HG > HH).

## Discussion

### Static tensile properties

The tensile modulus, *E*_*HFRP*_, and secondary tensile modulus, E*_HFRP_, of the HFRP specimens were calculated using a simple rule of mixtures:

1 $${E}_{HFRP}={E}_{F\left(IMS60\right)}{V}_{F\left(IMS60\right)}+{E}_{F\left(E{\text{-}}glass\right)}{V}_{F\left(E{\text{-}}glass\right)}+{E}_{M}{V}_{M}\quad \left(\text{for tensile modulus}\right),$$

and

2 $${{E}^{*}}_{HFRP}={E}_{GFRP}{V}_{GFRP} \quad \left(\text{for secondary modulus}\right),$$

where *E*_*F*(*IMS60*)_, *E*_*F*(*E-glass*)_, *E*_*M*_, and *E*_*GFRP*_ are the tensile moduli of the IMS60 fiber, E-glass fiber, matrix, and E-glass GFRP, respectively. *V*_*GFRP*_ is the volume fraction of E-glass GFRP. The volume fraction of each element is already known. The tensile modulus of the matrix is assumed to be *E*_*M*_ = 3.5 GPa. The tensile moduli of the CFRP and GFRP are obtained from the static tensile tests for the MC and MG specimens, and *E*_*CFRP*_ = 159 GPa and *E*_*GFRP*_ = 38 GPa, respectively. *E*_*F*(*IMS60*)_ and *E*_*F*(*E-glass*)_ are estimated from Eq. (1), and *E*_*F*(*IMS60*)_ = 277 GPa and *E*_*F*(*E-glass*)_ = 75 GPa.

The estimated tensile modulus (*E*_*cal*_) and secondary tensile modulus (E*_cal_) are shown in Table 3.

**Table 3:** Estimated tensile properties of the HFRP specimens.

	Described as	Tensile modulus *E*_*cal*_ (GPa)	Secondary tensile modulus E*_cal_ (GPa)	Tensile strength *σ*_*f.cal*_ (GPa)	Secondary fracture strength σ*_f.cal_ (GPa)	Failure strain *ε*_*f.cal*_ (GPa)	Secondary failure strain ε*_f.cal_ (GPa)
[(0_(IMS60)_)/(0_(E-glass)_)]_S_	HA	100	19	1.895	0.545	1.769	2.949
[(0_(E-glass)_)/(0_(IMS60)_)]_S_	HB	100	19	1.895	0.545	1.769	2.949
[(0_(E-glass)_)/(0_(IMS60)_)_2_]_S_	HC	120	12	2.275	0.361	1.769	2.949
[(0_(E-glass)_)/(0_(IMS60)_)_3_]_S_	HD	130	9	2.464	0.270	1.769	2.949
[(0_(E-glass)_)/(0_(IMS60)_)_5_]_S_	HE	139	6	2.651	0.180	1.769	2.949
[(0_(E-glass)_)_2_/(0_(IMS60)_)]_S_	HF	79	25	1.511	0.731	1.769	2.949
[(0_(E-glass)_)_3_/(0_(IMS60)_)]_S_	HG	69	29	1.317	0.825	1.769	2.949
[(0_(E-glass)_)_5_/(0_(IMS60)_)]_S_	HH	59	32	1.122	0.919	1.769	2.949

The term “hybrid ratio” was used to describe the hybrid effect. The hybrid ratio, *μ*, can be calculated as

3 $$\mu =\frac{{V}_{F(E{\text{-}}glass)}}{{V}_{F(IMS60)}+{V}_{F(E{\text{-}}glass)}}.$$

**Figure 3:**
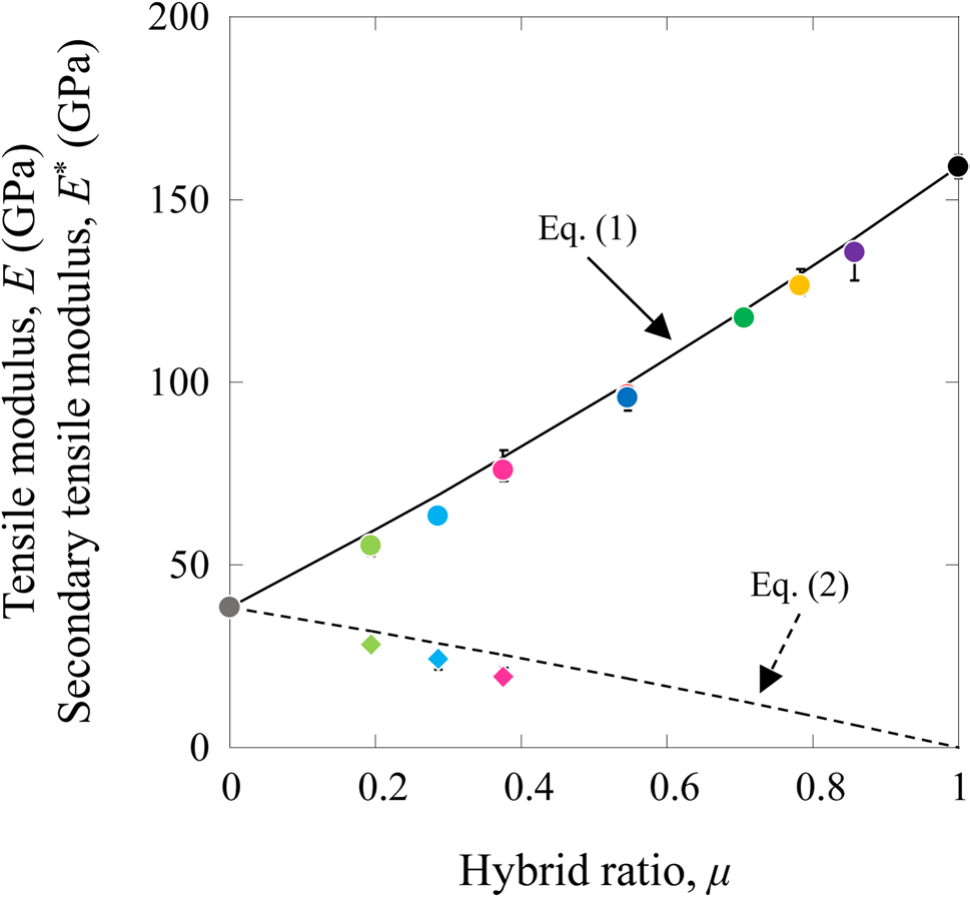
Relation between the tensile modulus and secondary tensile modulus of the HFRP specimens versus the hybrid ratio.

Figure 3 shows the relation between the estimated tensile modulus, *E*_*cal*_, and secondary tensile modulus, E*_cal_, of the HFRP specimens with the hybrid ratio, *μ*. The experimental results are also shown in this figure.

The experimental results of the tensile modulus and secondary tensile modulus of the HFRP specimens were in agreement with the rule of mixture prediction. Similar results of hybrid composites have been observed in the reported literature^17–19^.

The tensile strength, *σ*_*f(HFRP)*_, and secondary fracture strength, σ*_f(HFRP)_, of the HCFRP specimens were also calculated using the rule of mixtures:

4 $${\sigma }_{f\left(HFRP\right)}={\sigma }_{fF\left(IMS60\right)}{V}_{F\left(IMS60\right)}+\frac{{\sigma }_{fF\left(IMS60\right)}}{{E}_{F\left(IMS60\right)}}{E}_{F\left(E{\text{-}}glass\right)}{V}_{F\left(E{\text{-}}glass\right)}+\frac{{\sigma }_{fF\left(IMS60\right)}}{{E}_{F\left(IMS60\right)}}{E}_{M}{V}_{M} \left(\text{for tensile strength}\right),$$

5 $${\sigma }_{f\left(HFRP\right)}={\sigma }_{fF\left(E{\text{-}}glass\right)}{V}_{F\left(E{\text{-}}glass\right)}+\frac{{\sigma }_{fF\left(E{\text{-}}glass\right)}}{{E}_{F\left(E{\text{-}}glass\right)}}{E}_{M}{V}_{M} \left(\text{for tensile strength}\right),$$

and

6 $${\sigma }_{f\left(HFRP\right)}^{*}={\sigma }_{f\left(GFRP\right)}{V}_{GFRP} \left(\text{for secondary fracture strength}\right),$$

where *σ*_*fF*(*IMS60*)_, *σ*_*fF*(*E-glass*)_, and *σ*_*f*(*GFRP*)_ are the tensile strength values of the IMS60 fiber, E-glass fiber, and E-glass GFRP, respectively. The volume fraction and tensile modulus of each element are already known. The tensile strength values of the CFRP and GFRP are obtained from the static tensile tests for the MC and MG specimens, are *σ*_*f*(*CFRP*)_ = 3.023 GPa and *σ*_*f*(*GFRP*)_ = 1.109 GPa, respectively. *σ*_*fF*(*IMS60*)_ and *σ*_*fF*(*E-glass*)_ are estimated from Eqs. (4)–(6), and *σ*_*fF*(*IMS60*)_ = 5.279 GPa and *σ*_*fF*(*E-glass*)_ = 2.169 GPa.

The failure strain *ε*_*f(HFRP)*_ and secondary failure strain ε*_f(HFRP)_ of the HCFRP specimens were calculated using the following equations:

7 $${\varepsilon }_{f\left(HFRP\right)}=\frac{{\sigma }_{f\left(HFRP\right)}}{{E}_{HFRP}} \left(\text{for failure strain}\right),$$

and

8 $${{\varepsilon }^{*}}_{f\left(HFRP\right)}=\frac{{{\sigma }^{*}}_{f\left(HFRP\right)}}{{{E}^{*}}_{HFRP}} \left(\text{for secondary failure strain}\right).$$

The estimated tensile strength (*σ*_*f.cal*_), secondary fracture strength (σ*_f.cal_), failure strain (*ε*_*f.cal*_), and secondary failure strain (ε*_f.cal_) are shown in Table 3.

**Figure 4:**
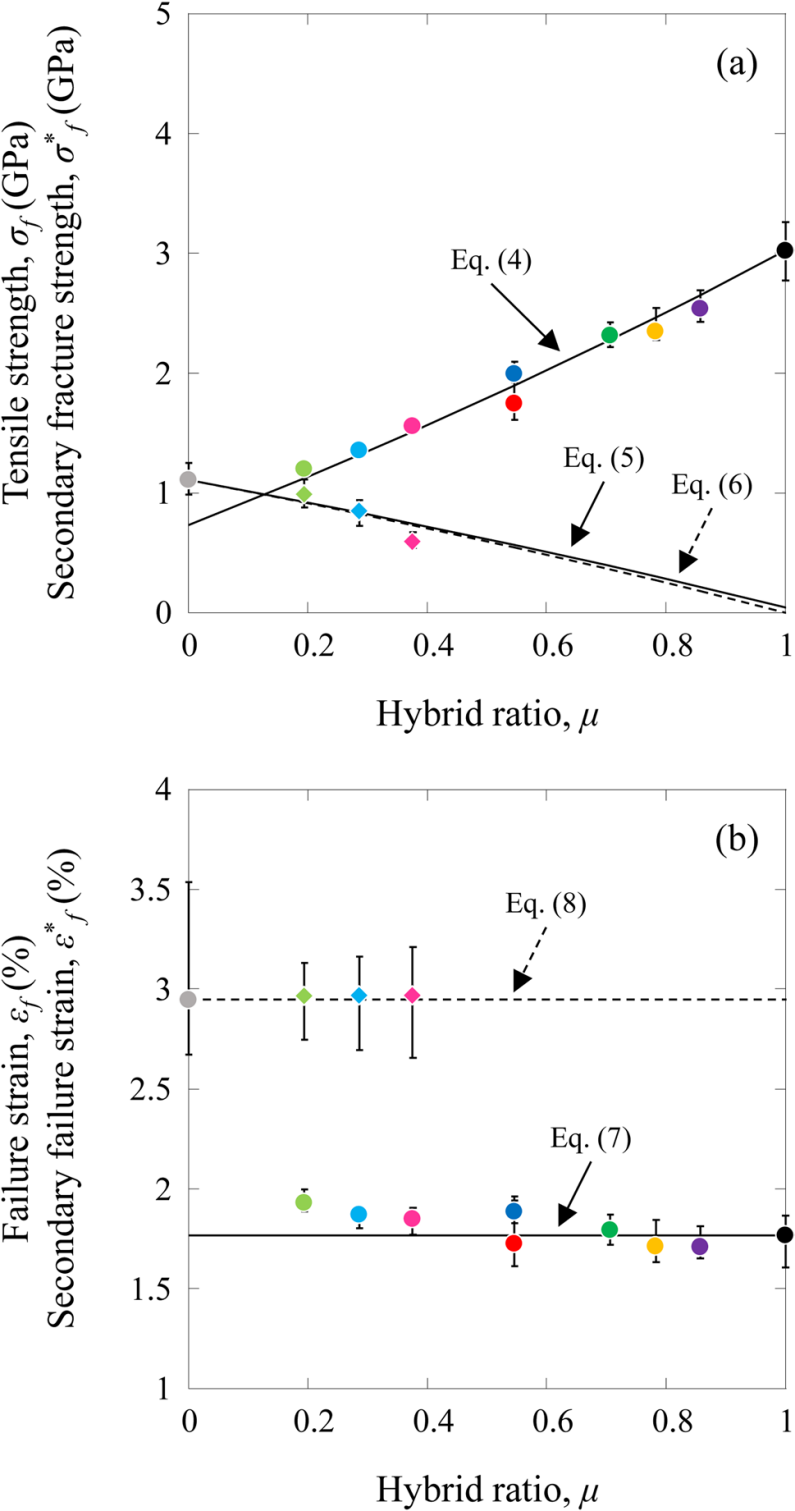
Relation between the tensile strength, secondary fracture strength, failure strain, and secondary failure strain of the HFRP specimens with the hybrid ratio. (**a**) Tensile strength and secondary fracture strength and (**b**) failure strain and secondary failure strain.

Figure 4 shows the relation between the estimated tensile strength, *σ*_*f.cal*_, secondary fracture strength, σ*_f.cal_, failure strain, *ε*_*f.cal*_, and secondary failure strain, ε*_f.cal_, of the HFRP specimens with the hybrid ratio. The experimental results are also shown in this figure.

The tensile strength values of the HB, HC, HF, HG, and HH specimens were higher than those of their predicted values. The secondary fracture strength values of the HG and HH specimens were higher than those of their predicted values. The secondary failure strains of the HF, HG, and HG specimens were similar to that of the MG specimen. However, the failure strains of the HB, HC, HF, HG, and HH specimens were also higher than that of the MC specimen. Similar results of hybrid composites have also been observed in some literature^20–23^.

There is an appreciable scattering of tensile strength for these composites. The statistical distribution of strength values is usually described by the Weibull equation^24–28^. The two-parameter Weibull distribution is given by

9 $${P}_{F}=1-\text{exp}\left[-{\left(\frac{{\sigma }_{f}}{{\sigma }_{0}}\right)}^{m}\right],$$

where *P*_*F*_ is the cumulative probability of failure of a composite at applied tensile strength *σ*_*f*_, *m* is the Weibull modulus (Weibull shape parameter) of the composite, and *σ*_0_ is a Weibull scale parameter (characteristic stress). The cumulative probability of failure, *P*_*F*_, under a particular stress is given by

10 $${P}_{F}=\frac{i}{n+1},$$

where *i* is the number of composite specimens that have broken at or below a stress level and *n* is the total number of composite specimens tested.

**Figure 5:**
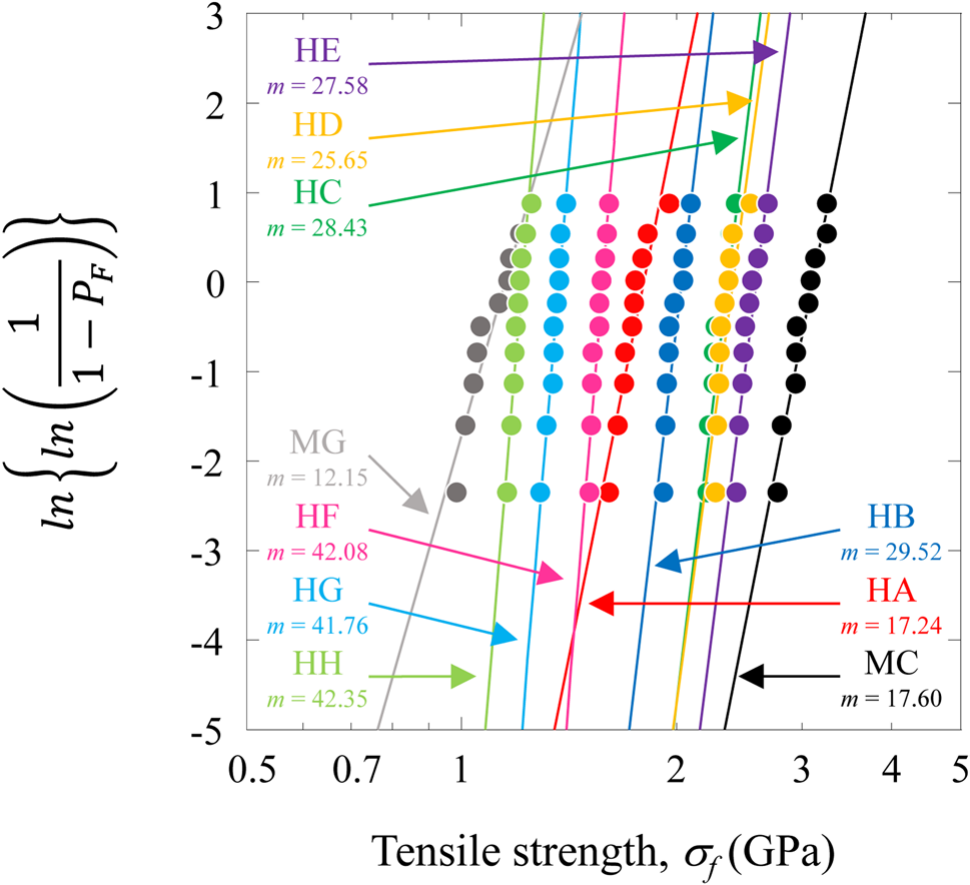
Weibull plots of the CFRP, GFRP, and HFRP specimens.

Figure 5 shows the Weibull plots of the MC, MG, and HFRP specimens. The Weibull moduli, *m*, for the MC and MG specimens were calculated to be 17.60 for the MC specimen and 12.15 for the MG specimen. *m* values for the HFRP specimens are shown in Table 2. *m* for the HA specimen was similar to that for the MC specimens, and *m* for the HB, HC, HD, HE, HF, HG, and HH specimens was higher than that for the MC and MG specimens. In particular, *m* for the HF, HG, and HH specimens showed higher values.

The results clearly show that the HFRP specimens, except for the HA specimen, improved the specimen Weibull moduli of tensile strength. The differences in *m* can be attributed to the nature and distribution of the flaws present in the specimens. It is well known that many defects, including voids, fiber breakage, and fiber misalignment^29^, are known to be introduced into these types of laminates during manufacturing and subsequent treatment. Outer high-ductility E-glass GFRP hybridization reduced the effects of the strength-limiting defects of IMS60 CFRP, which in turn, improved the Weibull moduli of HB, HC, HD, HE, HF, HG, and HH specimens. In contrast, the failures of the inner high-ductility E-glass GFRP hybridized HFRP specimen were predominantly initiated by outer IMS60 CFRP defects. Hybridization effects are less likely to appear. Consequently, the HA specimen did not improve the Weibull modulus of the IMS60 CFRP specimens.

**Figure 6:**
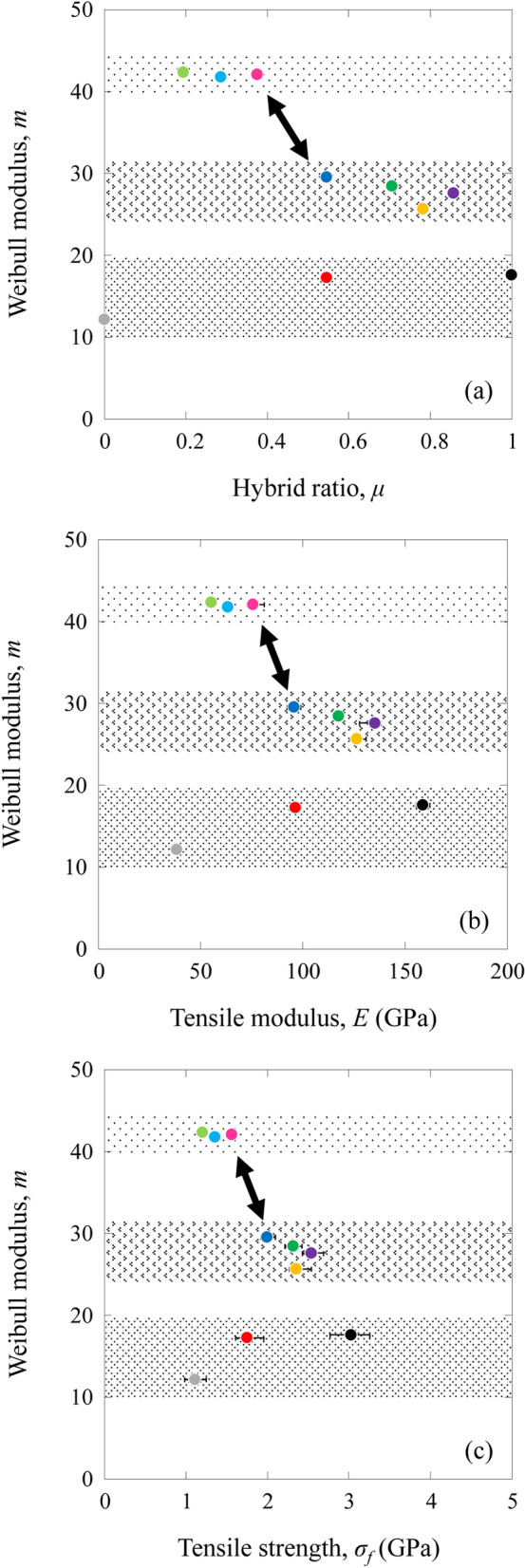
Weibull modulus of HFRP specimens as a function of hybrid ratio and tensile properties. (**a**) Hybrid ratio, (**b**) tensile modulus, and (**c**) tensile strength.

Figure 6 shows the *m* of the HFRP specimens as a function of the hybrid ratio and tensile properties (modulus and strength). There is a clear Weibull modulus transition value in the hybrid ratio, tensile modulus, and strength.

Axial tension failure in unidirectional CFRP and GFRP specimens led to fracture in the transverse direction at several points and was associated with longitudinal splitting of the composite^30^. A similar fracture morphology (longitudinal splitting) was observed for the MC, MG, and the HFRP specimens. The HFRP specimens failed by extensive longitudinal splitting, resulting in a brush-like fracture surface and suggesting that the higher-strength IMS60 fiber dominant the fracture behavior and increased fiber efficiency. A distinct difference in morphology between the HA, HB, HC, HD, and HE specimens and the HF, HG, and HH specimens was observed. The HF, HG, and HH specimens were covered with a large amount of E-glass GFRP layers. The principal transverse crack related to fiber fracture ran across the whole width and thickness of the IMS60 CFRP layers. A delamination crack was produced at the intersection of the transverse crack and propagated in the length direction near the interface between the IMS60 CFRP layers and the E-glass GFRP layers. The fractured surfaces of the HF, HG, and HH specimens showed large splitting surfaces with delaminated E-glass GFRP layers.

### Fatigue tensile properties

The *S*–*N* curves for the MC and MG specimens can be described by a power law model. The power law model^31^ is given by

11 $${\sigma }_{max}=a\cdot {\left({N}_{f}\right)}^{b},$$

where *a* and *b* are experimental constants. The least squares fitting of the fatigue trends with the power law model is illustrated in Fig. 2. The intercept, *a*, and slope, *b*, are calculated to be 3.292 and − 0.0356 for the MC specimen and 1.884 and − 0.0899 for the MG specimen, respectively.

The *S*–*N* curves of the CFRP- and GFRP-dominant behaviors in the HFRP specimens were calculated using the power law model of the CFRP and GFRP specimens and a simple rule of mixtures. The *S*–*N* curves of the CFRP-dominant HFRP behaviors were estimated to add the load acted on GFRP (different in the ratio of CFRP/GFRP) to the load of CFRP using the *S*–*N* curve of the MC specimen. The *S*–*N* curves of the GFRP-dominant HFRP behaviors were estimated to add the load acted on CFRP (different in the ratio of CFRP/GFRP) to the load of GFRP using the *S*–*N* curve of the MG specimen. The estimated results are also shown in Fig. 2.

**Figure 7:**
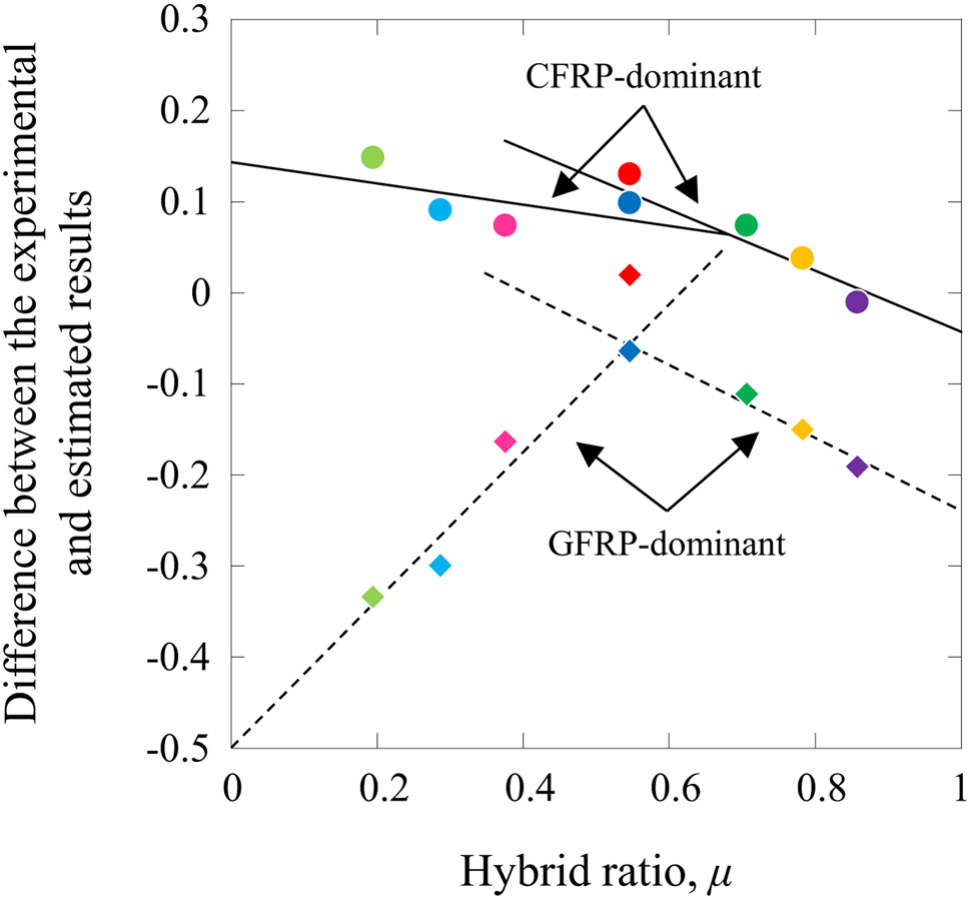
Difference between the experimental and estimated results as a function of the hybrid ratio.

Figure 7 shows the difference between the experimental and estimated results ((*X*_*exp*_ − *X*_*cal*_)/*X*_*cal*_, and *X* is the maximum applied stress for the same cycles) as a function of the hybrid ratio.

The CFRP-dominant behavior of the fatigue properties for the HA and HB specimens was higher than that of the estimated results. The GFRP-dominant behavior of the fatigue properties for the HA and HB specimens was higher and lower, respectively, than that of the estimated results. The fatigue properties of the HA specimen were approximately 5% higher than those of the HB specimen. For the HB, HC, HD, and HE specimens, the CFRP-dominant behavior of the fatigue properties decreased with increasing volume fraction of CFRP, and the GFRP-dominant behavior, which was lower than that of the estimated results, decreased with increasing volume fraction of CFRP. The CFRP-dominant behavior of the fatigue properties for the HE specimen was lower than that of the estimated results. On the other hand, for the HB, HF, HG, and HH specimens, the CFRP-dominant behavior of fatigue properties, which was higher than the estimated results, decreased with increasing volume fraction of CFRP. For the same specimens, the GFRP-dominant behavior, which was lower than the estimated results, increased with increasing volume fraction of CFRP.

**Figure 8:**
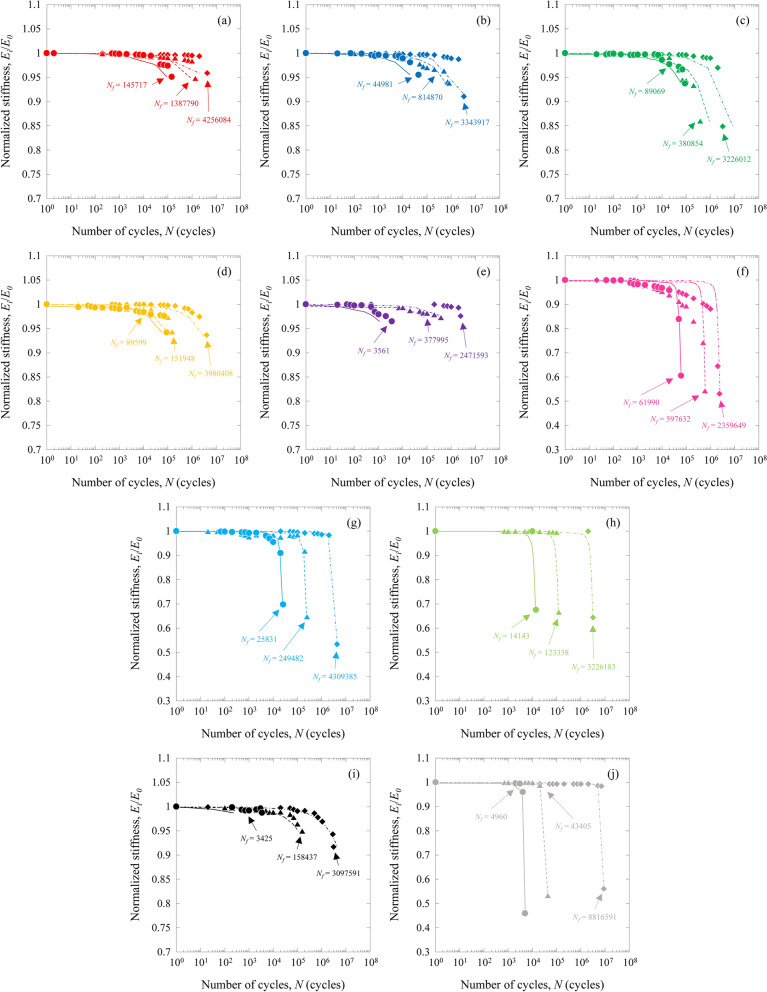
Stiffness reduction for the CFRP, GFRP, and HFRP specimens. (**a**) HA ([(0_(IMS60)_)/(0_(E-glass)_)]_S_), (**b**) HB ([(0_(E-glass)_)/(0_(IMS60)_)]_S_), (**c**) HC ([(0_(E-glass)_)/(0_(IMS60)_)_2_]_S_), (**d**) HD ([(0_(E-glass)_)/(0_(IMS60)_)_3_]_S_), (**e**) HE ([(0_(E-glass)_)/(0_(IMS60)_)_5_]_S_), (**f**) HF ([(0_(E-glass)_)_2_/(0_(IMS60)_)]_S_), (**g**) HG ([(0_(E-glass)_)_3_/(0_(IMS60)_)]_S_), (**h**) HH ([(0_(E-glass)_)_5_/(0_(IMS60)_)]_S_), (**i**) MC ((0_(IMS60)_)_4_), and (**j**) MG ((0_(E-glass)_)_4_).

Fatigue damage, such as matrix cracking and delamination, often results in a significant reduction in the modulus of composite laminates. Hence, it is crucial to develop an analytical model to describe the cumulative damage of composites due to fatigue based on apparent stiffness reduction^32–36^. Figure 8 shows apparent stiffness reduction during fatigue loading (low, middle, and high stress levels) for the mono CFRP and GFRP and HFRP specimens.

Most of the stiffness reduction occurred in the earlier stages of fatigue life, whereas the damage density increased steeply. The rate of stiffness degradation became very low as soon as the damage density reached a saturated value. The stiffness reduction trends of the HA, HB, HC, HD, and HE specimens were similar to those of the MC specimen. The stiffness reduction trends of the HF, HG, and HH specimens were similar to those of the MG specimen.

Stiffness reduction reflects the damaged state under fatigue cycles after the distribution of damage for the MC, MG, and HFRP specimens. The cumulative fatigue damage^30–34^ for the MC, MG, and HFRP specimens, *D*_*i*_, is defined as

12 $${D}_{i}=1-\frac{{E}_{i}}{{E}_{0}},$$

where *E*_*0*_ and *E*_*i*_ represent the apparent stiffness at the first cycle and the *i*-th cycle, respectively.

**Figure 9:**
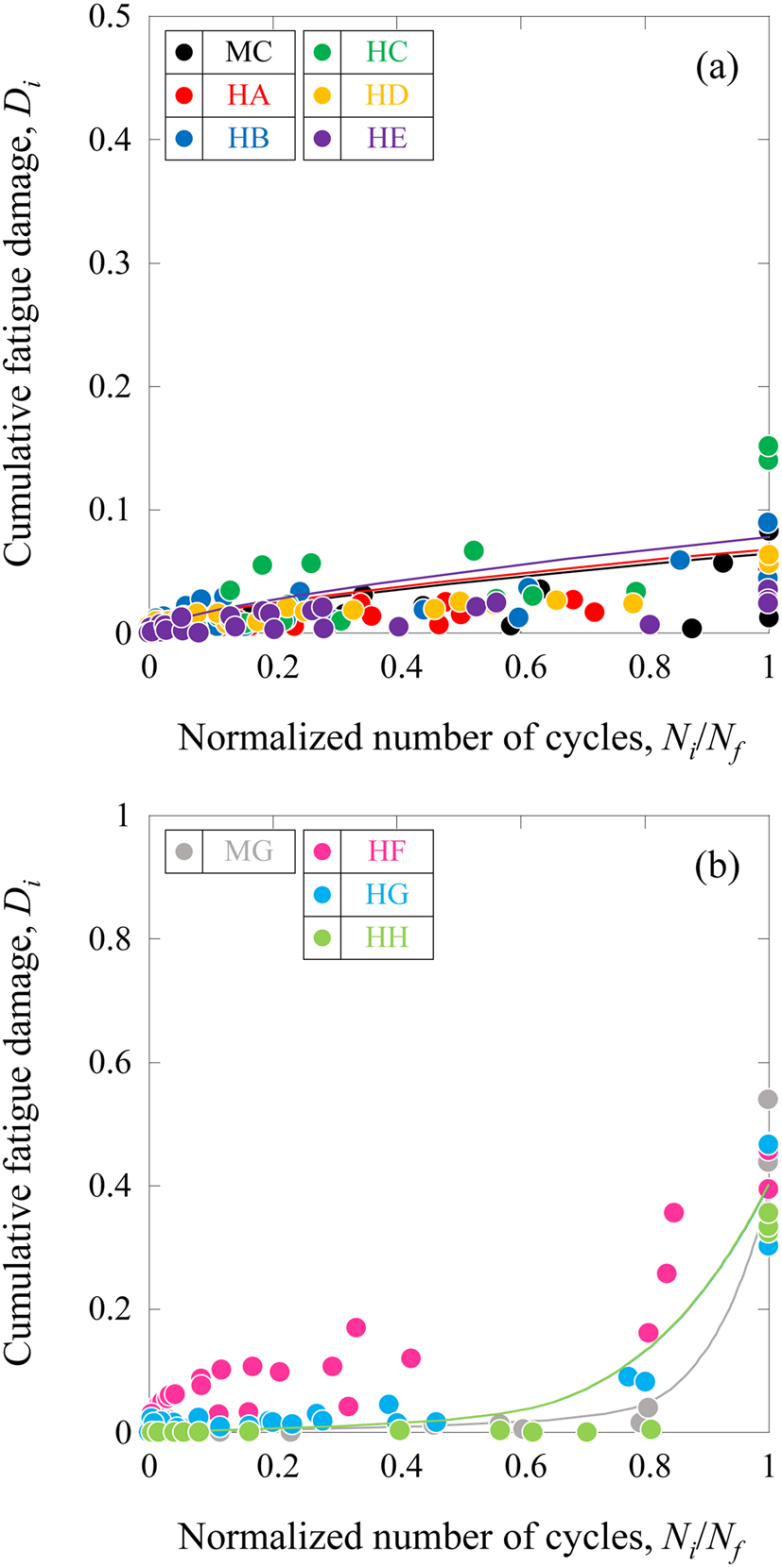
Cumulative fatigue damage for the CFRP, GFRP, and HFRP specimens as a function of the normalized number of cycles. (**a**) HA ([(0_(IMS60)_)/(0_(E-glass)_)]_S_), HB ([(0_(E-glass)_)/(0_(IMS60)_)]_S_), HC ([(0_(E-glass)_)/(0_(IMS60)_)_2_]_S_), HD ([(0_(E-glass)_)/(0_(IMS60)_)_3_]_S_), HE ([(0_(E-glass)_)/(0_(IMS60)_)_5_]_S_), and MC ((0_(IMS60)_)_4_), and (**b**) HF ([(0_(E-glass)_)_2_/(0_(IMS60)_)]_S_), HG ([(0_(E-glass)_)_3_/(0_(IMS60)_)]_S_), HH ([(0_(E-glass)_)_5_/(0_(IMS60)_)]_S_), and MG ((0_(E-glass)_)_4_).

Figure 9 shows the cumulative fatigue damage for the MC, MG, and HFRP specimens as a function of the normalized number of cycles, *N*_*i*_/*N*_*f*_ (*N*_*i*_ represents the *i-th* cycle), which is widely used in the literature^32–36^.

The cumulative fatigue damage, *D*_*i*_, for the MC, MG, and HFRP specimens increased with increasing *N*_*i*_/*N*_*f*_. For the MC, MG, and HFRP specimens, there exists a relationship between *D*_*i*_ and *N*_*i*_/*N*_*f*_, given by

13 $$\frac{{N}_{i}}{{N}_{f}}=C{\left({D}_{i}\right)}^{n}\left\{\frac{1-{\left(\frac{{e}^{{D}_{th}}}{{e}^{{D}_{i}}}\right)}^{{m}_{1}}}{1-{\left(\frac{{e}^{{D}_{i}}}{{e}^{{D}_{C}}}\right)}^{{m}_{2}}}\right\},$$

where *C*, *n*, *m*_*1*_, and *m*_*2*_ are experimental constants. *D*_*th*_ and *D*_*C*_ are the threshold and critical cumulative fatigue damages, respectively, and are assumed to be *D*_*th*_ = 0 and *D*_*C*_ = 1. The estimated relationship between *D*_*i*_ and *N*_*i*_/*N*_*f*_ is also shown in Fig. 9. The experimental results showed reasonable agreement with the estimated relation obtained from Eq. (13). The apparent stiffness reduction during fatigue loading for the MC, MG, and HFRP specimens was estimated using Eqs. (12) and (13), and these lines are also shown in Fig. 8. Here, the experimental results were found to agree well with the estimated lines. Therefore, Eq. (13) is effective for understanding the fatigue properties.

## Conclusions

The static and fatigue tensile properties of high-strength PAN-based (IMS60) and E-glass hybrid fiber-reinforced epoxy matrix composites (HFRP) were examined.

Under static loading, for the HA, HB, HC, HD, and HE specimens, the stress applied to the specimen was almost linearly proportional to the strain until failure. However, the tensile stress–strain curves of the HF, HG, and HH specimens showed a complicated shape (jagged trace). The tensile modulus and secondary tensile modulus for the HFRP specimens could be estimated from the rule of mixtures. The tensile strength values of the HB, HC, HF, HG, and HH specimens are higher than the values predicted by the rule of mixtures. The secondary fracture strength values of the HG and HH specimens are higher than those of the predicted values. The failure strains of the HB, HC, HF, HG, and HH specimens are higher than that of the MC specimen. The Weibull statistical distributions of the tensile strength were also examined. The Weibull moduli for the HB, HC, HD, HE, HF, HG, and HH specimens are higher than those for the MC and MG specimens. The Weibull modulus of the HA specimen is almost similar to that for the MC specimen.

Under fatigue loading, the fatigue properties of the HFRP specimens show CFRP-dominant behavior at high stress levels and GFRP-dominant behavior at low stress levels. The fatigue properties of the HFRP specimens increase with increasing volume fraction of CFRP (HE > HD > HC > HA > HB > HF > HG > HH). The fatigue properties of the HA specimen are higher than those of the HB specimen. For the HB, HC, HD, and HE specimens, the CFRP-dominant behavior of the fatigue properties decreases with increasing volume fraction of CFRP and the GFRP-dominant behavior decreases with increasing volume fraction of CFRP. On the other hand, for the HB, HF, HG, and HH specimens, the CFRP-dominant behavior of the fatigue properties decreases with increasing volume fraction of CFRP and the GFRP-dominant behavior increases with increasing volume fraction of CFRP. The stiffness reduction trends of the HA, HB, HC, HD, and HE specimens are similar to those of the MC specimen. The stiffness reduction trends of the HF, HG, and HH specimens are similar to those of the MG specimen.
